# Construction and validation of a predictive model for HBsAg loss in chronic hepatitis B patients treated with Peg-IFNα-2b

**DOI:** 10.3389/fcimb.2026.1865116

**Published:** 2026-07-28

**Authors:** Xinrui Ren, Xinyi Sheng, Zhengbinkelvin Wong, Yu Cui, Qi Zhang, Jiayi Song, Wanchun Zhu, Yueqiu Gao, Jianjun Hu, Lingying Huang

**Affiliations:** 1 Department of Liver Diseases, Shuguang Hospital Affiliated to Shanghai University of Traditional Chinese Medicine, Shanghai, China; 2 School of Traditional Chinese Medicine, Shanghai University of Traditional Chinese Medicine, Shanghai, China; 3 Laboratory of Cellular Immunity, Shanghai Key Laboratory of Traditional Chinese Clinical Medicine, Shuguang Hospital Affiliated to Shanghai University of Traditional Chinese Medicine, Shanghai, China; 4 Zhongshan Hospital, Fudan University, Shanghai, China; 5 Department of Infectious Diseases, Shanghai Pudong Hospital, Fudan University Pudong Medical Center, Pudong, Shanghai, China

**Keywords:** chronic hepatitis B (CHB), HBsAg loss, logistic regression, PEG-IFNα-2b, predictive model

## Abstract

**Background:**

Hepatitis B surface antigen (HBsAg) loss is the ideal treatment endpoint for chronic hepatitis B (CHB). Pegylated interferon alfa-2b (Peg-IFNα-2b) is currently the preferred drug for achieving HBsAg loss, but its efficacy varies significantly among different patients.

**Methods:**

This multicenter, retrospective real-world study enrolled CHB patients treated with Peg-IFNα-2b for ≥48 weeks. Patients were divided into cured and uncured groups based on week-48 HBsAg status and randomly assigned to training and validation cohorts (7:3). Logistic regression identified independent predictors of HBsAg loss and constructed a predictive model. The model’s discriminative ability, calibration, and clinical applicability were evaluated using receiver operating characteristic (ROC) curve analysis, calibration curves, Hosmer–Lemeshow test, and decision analysis curve (DCA) analysis.

**Results:**

Among 859 patients, 133 (15.5%) achieved HBsAg loss at week 48. Multivariate analysis identified combination therapy with traditional Chinese medicine (TCM) (OR = 3.83), lower baseline HBsAg (OR = 0.32), lower baseline aspartate aminotransferase (AST) (OR = 0.97), and entecavir (ETV) regimen (OR = 0.33) as independent predictors. The model demonstrated strong discrimination and calibration performance, with AUCs of 0.884 in the training cohort and 0.866 in the validation cohort. Logit(P)=-0.87-1.12*HBsAg(log10)+1.34*group-0.03*AST-1.1×ETV. Note: group (1=no combination with TCM, 2=combination with TCM), ETV (0=non-ETV antiviral regimen, 1=ETV antiviral regimen).

**Conclusion:**

The combination of TCM therapy, low baseline HBsAg levels, lower baseline AST levels, and non-ETV antiviral regimens were significant predictors of HBsAg loss at week 48 in CHB patients receiving 48-week Peg-IFNα-2b treatment. The predictive model we developed demonstrated high accuracy and potential clinical utility.

**Key points:**

This study focused on the establishment and validation of a clinical prediction model for HBsAg loss based on a large two-center cohort including 859 patients with CHB. The model was constructed using rigorous statistical methods and further evaluated through both internal and external validation. The results demonstrated good discrimination, calibration, and clinical net benefit, suggesting that the model may serve as a practical tool to help clinicians estimate the probability of HBsAg loss and facilitate individualized antiviral treatment strategies.

## 1 Introduction

CHB is a chronic liver disease caused by persistent infection with the hepatitis B virus (HBV). It is a leading cause of liver cirrhosis, hepatocellular carcinoma (HCC), and liver-related disease progression and mortality worldwide. According to World Health Organization (WHO) data, the global HBsAg prevalence rate was 3.8% in 2019, with approximately 1.5 million new HBV infections that year. There are currently about 296 million people living with chronic HBV infection globally, and around 820,000 deaths are attributed to HBV-related conditions (Chinese Society of Hepatology, Chinese Medical Association and Chinese Society of Infectious Diseases, Chinese Medical Association, 2022). Due to persistent transmission in some regions and limited access to treatment for infected individuals, an estimated 63 million new cases of chronic HBV infection and 17 million HBV-related deaths are projected between 2015 and 2030 (Nayagam et al., 2016). Multiple international and domestic clinical guidelines consistently recommend that achieving HBsAg loss and clinical cure can significantly reduce the risks of decompensated liver disease, HCC, and liver-related mortality, and is considered the ideal endpoint for antiviral therapy (Fu et al., 2025).

Currently, the clinically approved antiviral regimens for CHB primarily consist of two major categories: pegylated interferon (PEG-IFN) and nucleos(t)ide analogues (NAs), both of which can inhibit HBV replication and delay disease progression (Tout et al., 2020). NAs can suppress viral replication and restore adaptive immunity over the long term, but they are ineffective in inhibiting the transcriptional activity of covalently closed circular DNA (cccDNA), leaving patients at risk of HCC (Bourliere et al., 2017). In contrast, PEG-IFNα significantly enhances HBsAg loss by boosting innate immunity, activating T-cell-mediated immune responses, suppressing HBV protein expression, and reducing intrahepatic cccDNA reservoirs—a combination of effects that makes it the cornerstone strategy for achieving HBsAg loss (Tan et al., 2024). However, significant variations exist in patients’ responses to PEG-IFNα therapy, necessitating the identification of key predictive factors and the development of reliable predictive models for precise patient selection and individualized treatment optimization.

Based on the aforementioned research background, this study was conducted as a retrospective real-world cohort, enrolling CHB patients treated with interferon in the Hepatology Department of Shanghai Shuguang Hospital Affiliated to Shanghai University of Traditional Chinese Medicine and the Infectious Diseases Department of Tongren Hospital Affiliated to Shanghai Jiao Tong University School of Medicine, both in outpatient and inpatient settings. A predictive model for HBsAg loss at week 48 was constructed and validated through systematic analysis of independent predictors, providing evidence-based medical support for precise clinical cure of CHB.

## 2 Materials and methods

### 2.1 Method

This multicenter, retrospective, real-world study involved CHB patients with either initial or prior treatment from Shanghai Shuguang Hospital affiliated to Shanghai University of Traditional Chinese Medicine and Tongren Hospital affiliated to Shanghai Jiao Tong University School of Medicine. All patients received Peg-IFNα-2b monotherapy or combination therapy with NAs, with a follow-up period of no less than 48 weeks. The primary endpoint of this study was HBsAg loss at week 48 of PEG-IFN therapy. Serum HBsAg was quantitatively measured by chemiluminescence immunoassay, and the lower limit of detection (LOD) of the assay kit was 0.05 IU/mL. HBsAg loss at week 48 was defined as serum HBsAg concentration <0.05 IU/mL at this time point.

Patients were differentiated into five TCM syndromes (damp-heat in liver-gallbladder, liver-stagnation and spleen-deficiency, liver-kidney yin deficiency, spleen-kidney yang deficiency, and static blood obstructing collaterals) in accordance with the Expert Consensus on Integrated Traditional Chinese and Western Medicine Diagnosis and Treatment of Chronic Hepatitis B. Corresponding modified herbal formulas were administered, including Yinchenhao Decoction, Xiaoyao San, Yiguan Jian, Fuzi Lizhong Decoction and Gexia Zhuyu Decoction.

Patients in the combined TCM group received syndrome-differentiated herbal decoctions alongside Peg-IFN antiviral therapy. Herbal treatment was initiated simultaneously with interferon and administered continuously for at least 6 months. The herbal prescriptions were adjusted dynamically by the attending physicians according to liver function, virological indicators and evolving clinical syndromes.

Inclusion criteria: 1. Age 18–80 years (inclusive), no gender restriction; 2. Meeting the diagnostic criteria for CHB; 3. Having received PEG-IFNα-2b treatment for ≥48 weeks; 4. Complete clinical records and follow-up data.

Exclusion criteria: 1. Patients with concurrent infections by hepatitis A, C, D, E viruses, or non-hepatotropic viruses, such as Epstein-Barr virus (EBV), cytomegalovirus (CMV), or human immunodeficiency virus (HIV); 2. Patients with concurrent autoimmune hepatitis, primary sclerosing cholangitis, Wilson’s disease, alpha-1 antitrypsin deficiency, or suspected/confirmed history of alcohol or drug abuse; 3. Patients with concurrent decompensated cirrhosis, HCC, or other chronic liver diseases; 4. Patients with concurrent severe primary circulatory, digestive, respiratory, urinary, or hematologic disorders, or other life-threatening conditions and psychiatric diseases; 5. Pregnant and lactating women; 6. Patients with incomplete medical records or insufficient serological, biochemical, or imaging test results.

### 2.2 Flowchart of patient selection and study design

**Figure 1:**
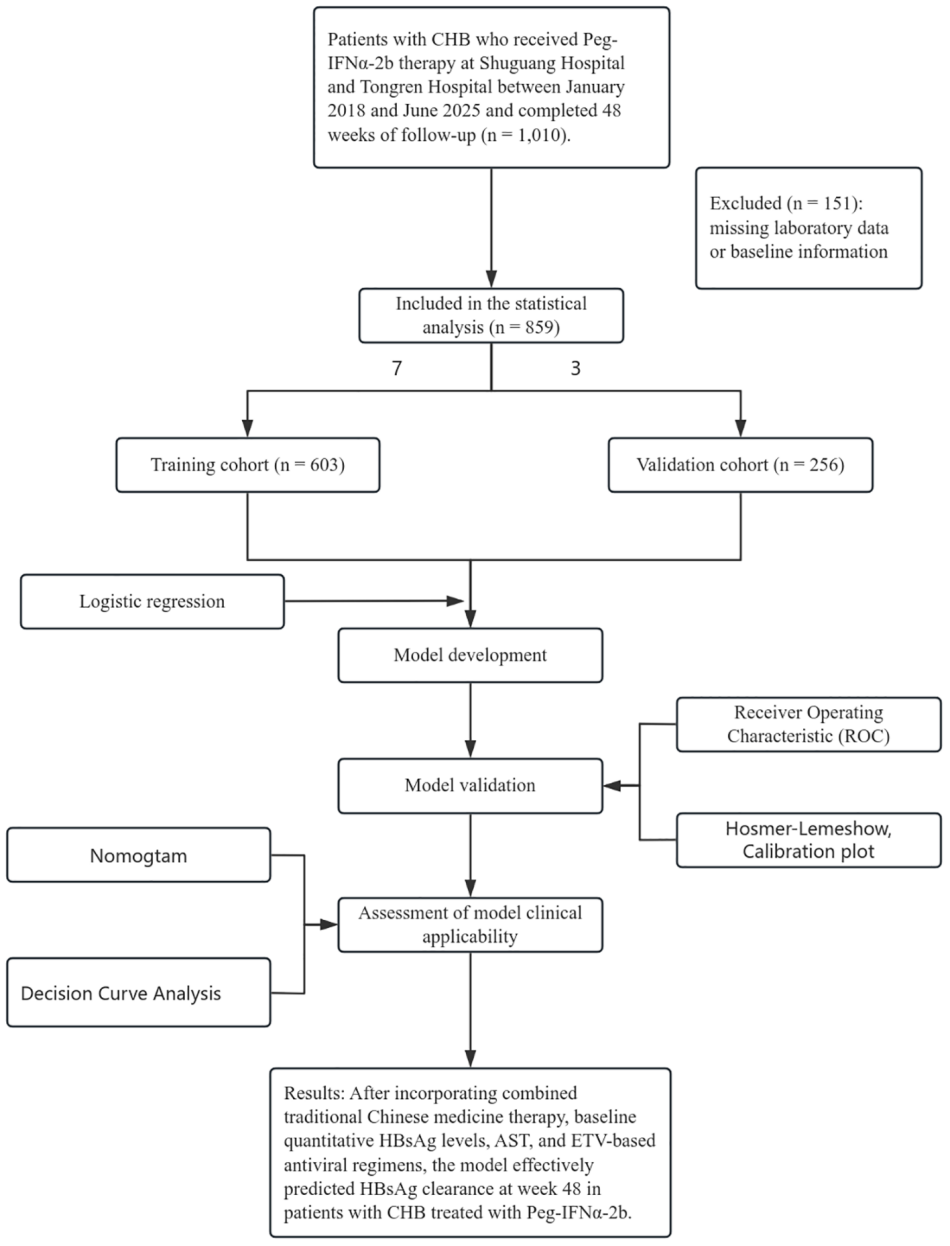
Flowchart of the study design.

After screening, a total of 1,010 CHB patients who received interferon therapy in the Hepatology Department of Shanghai Shuguang Hospital (affiliated to Shanghai University of Traditional Chinese Medicine) and the Infectious Diseases Department of Tongren Hospital (affiliated to Shanghai Jiao Tong University School of Medicine) from January 2018 to June 2025 were initially enrolled. Among them, 151 patients were excluded due to missing follow-up data or incomplete key clinical trial data. Ultimately, 859 CHB patients were included in the statistical analysis. The flowchart for the screening and grouping of study subjects is shown in **Figure 1**.

First, based on the inclusion and exclusion criteria, we ultimately enrolled 859 patients and randomly divided them into a training cohort (603 cases) and a validation cohort (256 cases) in a 7:3 ratio. In the training cohort, univariate and multivariate logistic regression analyzes were performed to screen for independent factors associated with HBsAg loss. Subsequently, predictive models were constructed based on the selected predictors, and column-line charts were further developed to visualize the probability of HBsAg loss.

### 2.3 Statistical analysis

Statistical analysis was performed using SPSS 27.0 and R 4.3.3. Categorical variables were presented as frequency and percentage, with intergroup comparisons conducted using the chi-square test. Continuous variables were expressed as mean ± 95% confidence interval (CI); normally distributed data were analyzed using the independent samples t-test, while non-normally distributed data were compared using the Mann–Whitney U test. Univariate and multivariate logistic regression analyzes were employed to identify independent predictors of HBsAg loss and to construct a predictive model for HBsAg loss. The discriminant capacity of the model was evaluated using the ROC curve, while the calibration of the model was assessed using the calibration curve and the Hosmer–Lemeshow test. The clinical applicability of the model was evaluated through DCA. A P-value <0.05 was considered statistically significant.

## 3 Results

### 3.1 Research characteristics

This study enrolled a total of 859 patients with CHB, among whom 133 patients achieved HBsAg loss at 48 weeks (15.5%), while 726 patients did not achieve loss (84.5%). Statistical analysis revealed statistically significant differences between the loss group and the non-loss group in terms of the use of TCM, antiviral treatment regimens, alanine aminotransferase (ALT), AST, baseline HBsAg quantitative levels and baseline HBeAg status (P<0.05), as shown in **Table 1**. No statistically significant differences were observed in baseline characteristics between the training cohort (n=603) and the validation cohort (n=256) (P>0.05), indicating good comparability of baseline characteristics between the two cohorts, as shown in **Table 2**. To further evaluate the inter-center homogeneity of TCM intervention, we compared the TCM application rate and TCM syndrome distribution between the two participating centers (**Supplementary Table 1**). The overall proportion of patients receiving combined TCM therapy was 46.4% in the entire cohort, with a rate of 47.3% in the TCM hospital (Center A) and 41.4% in the Western general hospital (Center B). No significant difference in TCM usage was found between the two centers (P = 0.215). The distribution of five TCM syndrome subtypes was highly consistent and comparable between the two centers (P = 0.935), with liver-stagnation and spleen-deficiency syndrome consistently predominant in both clinical settings.

**Table 1:** Baseline characteristics of all participants.

Variable	Total patients	Loss group	Non-loss group	*P value*
	N=859	N=133	N=726	
Age	39.27 (38.65, 39.89)	40.29 (38.74, 41.83)	39.08 (38.41, 39.76)	0.166
Gender (Male)	712(82.9%)	107(80.5%)	605(83.3%)	0.417
Family History	262(30.5%)	37(27.8%)	225(31%)	0.465
MAFLD	30(3.5%)	3(2.3%)	27(3.7%)	0.398
Drinking history	8(0.9%)	1(0.8%)	7(1.0%)	0.815
Smoking history	7(0.8%)	1(0.8%)	6(0.8%)	0.930
Combined traditional Chinese medicine therapy	399 (46.4%)	96(72.7%)	303(41.7%)	0.000
Antiviral drug				0.043
ETV	289 (33.6%)	36 (27.1%)	253(34.8%)	
TAF	148 (17.2%)	16(12%)	132(18.2%)	
TDF	110 (12.8%)	17(12.8%)	93(12.8%)	
3TC	2(0.2%)	0 (0%)	2(0.3%)	
TMF	13 (1.5%)	2(1.5%)	11(1.5%)	
Peg-IFN monotherapy	297 (34.6%)	62 (46.6%)	235 (32.4%)	
WBC	4.62 (4.5, 4.73)	4.63 (4.31, 4.94)	4.61 (4.49, 4.74)	0.924
RBC	4.87 (4.84, 4.91)	4.85 (4.76, 4.94)	4.88 (4.84, 4.92)	0.597
PLT	172.68 (168.7, 176.66)	180.47 (170.07, 190.86)	171.26 (166.95, 175.56)	0.100
Hb	146.46 (145.3, 147.63)	144.81 (141.77, 147.84)	146.77 (145.51, 148.03)	0.231
Neutrophile granulocyte	2.69 (2.54, 2.84)	2.66 (2.41, 2.91)	2.7 (2.53, 2.86)	0.871
FPG	5.16 (5.09, 5.23)	5.18 (4.99, 5.38)	5.15 (5.07, 5.23)	0.771
ALT	49.56 (45, 54.12)	33.68 (29.32, 38.04)	52.47 (47.16, 57.78)	0.003
AST	41.03 (38.54, 43.51)	30.96 (28.86, 33.07)	42.87 (39.97, 45.77)	0.001
HBsAg (log10)	2.53 (2.44, 2.62)	1.12 (0.91, 1.33)	2.79 (2.7, 2.87)	0.000
HBeAg (positive)	232(27%)	21(15.8%)	211(29.1%)	0.002
HBV DNA (>50 IU/ml)	50(5.8%)	6(4.5%)	44(6.1%)	0.483

Data are presented as mean (95% confidence interval) for continuous variables and n (%) for categorical variables.Abbreviations: 3TC, Lamivudine; ALT, Alanine aminotransferase; AST, Aspartate aminotransferase; FPG, Fasting plasma glucose; Hb, Hemoglobin; HBeAg, Hepatitis B e antigen; HBsAg, Hepatitis B surface antigen; HBV DNA, Hepatitis B virus deoxyribonucleic acid; MAFLD, Metabolic dysfunction-associated fatty liver disease; PLT, Platelet; RBC, Red blood cell; TAF, Tenofovir alafenamide; TDF, Tenofovir disoproxil fumarate; TMF, tenofovir amibufenamide; ETV, Entecavir; WBC, White blood cell.

**Table 2:** Baseline characteristics of patients in the training and validation cohorts.

Variable	Training set	Validation set	P value
	N=603	N=256	
Age	39.33 (38.59, 40.06)	39.14 (38.02, 40.26)	0.788
Gender (Male)	498 (82.6%)	214 (83.6%)	0.720
Family History	194 (32.2%)	68 (26.6%)	0.102
MAFLD	24 (4.0%)	6 (2.3%)	0.232
Drinking history	5 (0.8%)	3 (1.2%)	0.632
Smoking history	5 (0.8%)	2 (0.8%)	0.943
Combined traditional Chinese medicine therapy	280 (46.4%)	119 (46.5%)	0.989
Antiviral drug			0.053
ETV	215 (35.7%)	74 (28.9%)	
TAF	109 (18.1%)	39 (15.2%)	
TDF	76 (12.6%)	34 (13.3%)	
3TC	0 (0.0%)	2 (0.78%)	
TMF	8 (1.3%)	5 (2.0%)	
Peg-IFN monotherapy	195(32.3%)	102(39.8%)	
WBC	4.56 (4.42, 4.7)	4.74 (4.53, 4.96)	0.160
RBC	4.86 (4.82, 4.91)	4.9 (4.83, 4.97)	0.372
PLT	170.47 (165.78, 175.15)	177.9 (170.38, 185.42)	0.093
Hb	146 (144.59, 147.41)	147.55 (145.5, 149.61)	0.230
Neutrophile granulocyte	2.67 (2.47, 2.87)	2.74 (2.57, 2.9)	0.691
FPG	5.14 (5.06, 5.21)	5.21 (5.05, 5.38)	0.361
ALT	50.54 (44.75, 56.33)	47.24 (40.28, 54.21)	0.516
AST	42.33 (39.09, 45.58)	37.95 (34.6, 41.29)	0.113
HBsAg (log10)	2.56 (2.45, 2.66)	2.46 (2.29, 2.63)	0.340
HBeAg (positive)	166 (27.5%)	66 (25.8%)	0.598
HBV DNA (>50 IU/ml)	35 (5.8%)	15 (5.9%)	0.975

Data are presented as mean (95% confidence interval) for continuous variables and n (%) for categorical variables.

### 3.2 Single-factor and multi-factor logistic regression analysis

A univariate logistic regression analysis was first performed to examine the relationship between each variable and HBsAg loss. The results demonstrated that combined TCM therapy, baseline HBsAg level, ALT, AST, and ETV antiviral regimen were significantly associated with HBsAg loss. Subsequently, these variables were included in a multivariate logistic regression model, which identified combined TCM therapy, baseline HBsAg level, AST, and ETV antiviral regimen as independent predictors of HBsAg loss (**Table 3**). Specifically, combined TCM therapy significantly increased the rate of HBsAg loss (OR = 3.83, 95% CI: 2.13–6.87). A history of ETV use was negatively associated with HBsAg loss (OR = 0.33, 95% CI: 0.17–0.63). Higher baseline HBsAg levels (log10 IU/mL) were negatively associated with HBsAg loss (OR = 0.32, 95% CI: 0.25–0.42), higher baseline AST levels were negatively associated (OR = 0.97, 95% CI: 0.95–0.99).

**Table 3:** Univariate and multivariate logistic regression analysis of factors related to HBsAg Loss at 48 weeks of Peg-IFNα-2b treatment.

Variable	Univariate logistic regression			Multivariate logistic regression		
	OR	95%CI	P	OR	95%CI	P
Age	1.00	0.98-1.03	0.916			
Gender (Male)	1.06	0.59-1.92	0.837			
Family History	0.76	0.46-1.26	0.288			
MAFLD	0.52	0.12-2.25	0.383			
Drinking history	-–-	-–-	-–-			
Smoking history	-–-	-–-	-–-			
Combined traditional Chinese medicine treatment (group)	2.87	1.77-4.65	0.000	3.83	2.13-6.87	0.000
Antiviral therapy						
ETV	0.39	0.22-0.70	0.002	0.33	0.17-0.63	0.001
TAF	0.64	0.33-1.22	0.175			
TDF	0.90	0.46-1.78	0.769			
3TC	-–-	-–-	-–-			
TMF	0.57	0.07-4.78	0.606			
WBC	0.99	0.87-1.12	0.846			
RBC	1.02	0.68-1.54	0.915			
PLT	1.00	1.00-1.01	0.069			
Hb	0.99	0.98-1.01	0.334			
Neutrophile granulocyte	0.99	0.89-1.10	0.835			
FPG	1.06	0.85-1.32	0.621			
ALT	0.98	0.97-0.99	0.001			
AST	0.99	0.95-0.99	0.000	0.97	0.95-0.99	0.002
HBsAg (log10)	0.38	0.31-0.47	0.000	0.32	0.25-0.42	0.000
HBeAg (positive)	0.64	0.37-1.11	0.11			
HBV DNA (>50 IU/ml)	0.74	0.26-2.16	0.586			

Data are presented as mean (95% confidence interval) for continuous variables and n (%) for categorical variables.

Drinking history, smoking history and 3TC were excluded from the final multivariate model due to quasi-complete separation (zero or near-zero cell counts in the contingency table).

### 3.3 Prediction model construction

A predictive model for HBsAg loss was constructed based on stepwise forward logistic regression analysis (AIC = 348.46).


Y=-0.87−1.12×HBsAg(log10)+1.34×group-0.03×AST-1.1×ETV

$$P=e^{y}/\left(1+e^{y}\right)$$

**Figure 2:**
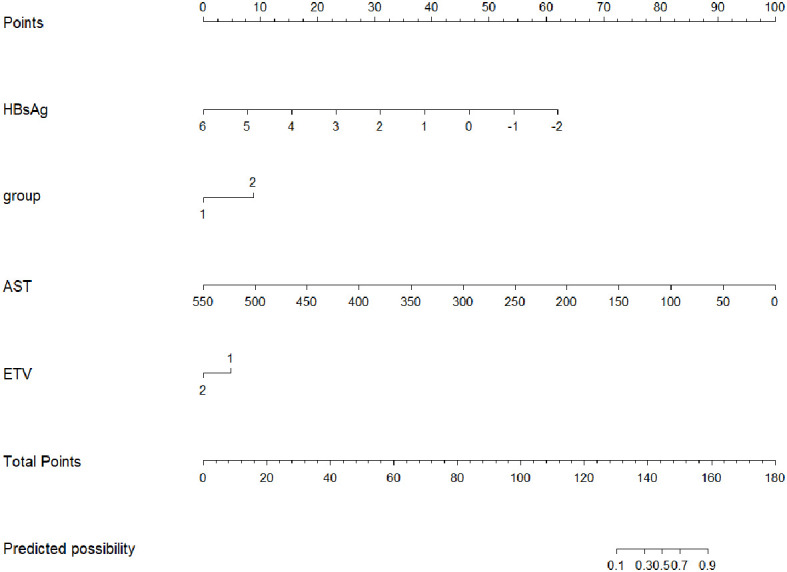
Column diagram of the prediction model. AST, Aspartate aminotransferase; HBsAg, Hepatitis B surface antigen; ETV, Entecavir.

Note: group (1=no combination with TCM, 2=combination with TCM), ETV (0=non-ETV antiviral regimen, 1=ETV antiviral regimen). The aforementioned predictive model is presented in the form of a column chart to quantitatively predict the probability of HBsAg loss in CHB patients after 48 weeks of Peg-IFNα-2b treatment (**Figure 2**).

### 3.4 Validation of the prediction model

**Figure 3:**
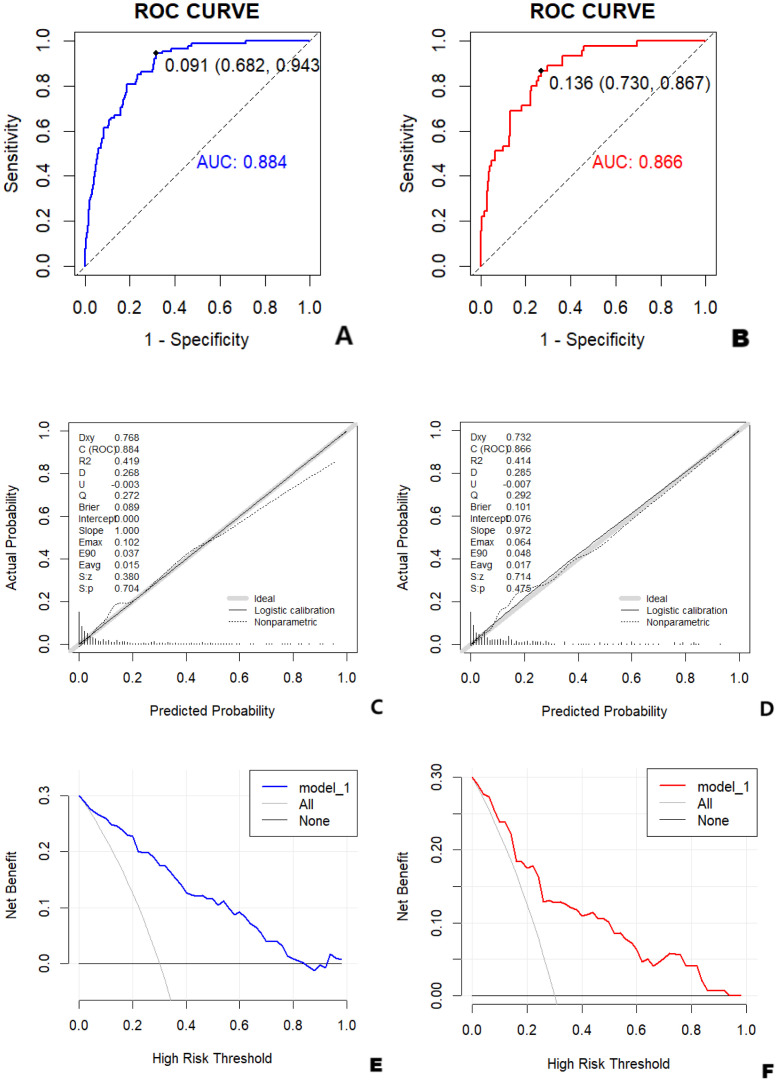
ROC curves, calibration curves, and decision curve analysis of the predictive model. **(A, B)** ROC curves for the training set **(A)** and validation set **(B)**. **(C, D)** Calibration curves for the training set **(C)** and validation set **(D)**. **(E, F)** DCA for the training set **(E)** and validation set **(F)**. ROC, receiver operating characteristic; DCA, decision analysis curve.

ROC curves were used to evaluate the discriminative ability of the predictive model. The x-axis represented specificity, and the y-axis represented sensitivity. ROC curves were plotted for both the training and validation cohort, yielding areas under the curve (AUC) of 0.884 (95% CI: 0.852–0.916) and 0.866 (95% CI: 0.814–0.918), respectively, indicating that the predictive model demonstrates good discriminative ability (**Figures 3A, B**). Comprehensive discriminative indicators of the two cohorts were further quantified and summarized in **Table 4**. In the training cohort, the sensitivity was 0.943 (95% CI: 0.872–0.981), specificity 0.682 (95% CI: 0.639–0.722), accuracy 0.720 (95% CI: 0.682–0.755), positive predictive value (PPV) 0.336 (95% CI: 0.277–0.399), and negative predictive value (NPV) 0.986 (95% CI: 0.968–0.995). For the validation cohort, the sensitivity reached 0.933 (95% CI: 0.817–0.986), specificity 0.621 (95% CI: 0.552–0.687), accuracy 0.676 (95% CI: 0.615–0.733), PPV 0.344 (95% CI: 0.261–0.436), and NPV 0.978 (95% CI: 0.936–0.995). Both cohorts presented high sensitivity and extremely high NPV, indicating a low likelihood of false-negative rate and reliable capacity to rule out patients with minimal possibility of HBsAg loss. Nevertheless, moderate specificity and relatively low PPV suggested a non-negligible false-positive rate.

**Table 4:** Comprehensive discriminative performance metrics of the prediction model in training and internal validation cohorts.

Index	Train	Valid
AUC	0.884(0.852, 0.916)	0.866(0.814, 0.918)
Sensitivity	0.943(0.872, 0.981)	0.933(0.817, 0.986)
Specificity	0.682(0.639, 0.722)	0.621(0.552, 0.687)
Accuracy	0.720(0.682, 0.755)	0.676(0.615, 0.733)
PPV	0.336(0.277, 0.399)	0.344(0.261, 0.436)
NPV	0.986(0.968, 0.995)	0.978(0.936, 0.995)

Data are presented as mean (95% confidence interval).

PPV, Positive predictive value; NPV, Negative predictive value; AUC, area under the curve.

The calibration of the model was evaluated using calibration curves and the Hosmer–Lemeshow test. The Hosmer–Lemeshow test was performed on both the training and validation cohorts, with P-values of 0.936 and 0.539, respectively, showing no significant deviation. The calibration curve results indicated good consistency between the predicted values and the actual observed values (**Figures 3C, D**).

Furthermore, DCA demonstrated that decision-making based on this model yielded higher net clinical benefits across a broad clinical decision interval, with consistent trends across cohorts, indicating good stability and reproducibility of the model in independent samples (**Figures 3E, F**).

## 4 Discussion

CHB is a significant global public health issue. Peg-IFNα-2b exhibits dual antiviral and immunomodulatory effects. It induces host cells to produce antiviral proteins to suppress viral replication, while simultaneously activating innate immunity and reconstructing adaptive immunity to break the immune tolerance state of the host. It is currently one of the most promising single-agent treatment regimens for achieving clinical cure (HBsAg loss) (Chinese Society of Hepatology, Chinese Medical Association and Chinese Society of Infectious Diseases, Chinese Medical Association, 2022; Zhang et al., 2024). Previous studies have shown that the spontaneous HBsAg loss rate in CHB patients is only approximately 1.17%; the HBsAg loss rate after NAs therapy is about 1%-3%; whereas the HBsAg loss rate after PEG-IFNα-2b therapy can reach 2.1%-20%, demonstrating its advantage in pursuing clinical cure. However, significant variations exist in patients’ responses to PEG-IFNα-2b therapy (Song et al., 2021; Yang et al., 2022). Based on this, the present study primarily evaluated the efficacy endpoint of HBsAg loss at 48 weeks, systematically assessed the factors influencing HBsAg loss through univariate and multivariate analyzes, and ultimately identified four key predictive variables: combination with TCM, baseline HBsAg quantitative level, AST, and ETV medication history.

### 4.1 Combined TCM therapy

This study found that in CHB patients treated with PEG-IFNα, the combination of TCM therapy during treatment significantly improved the HBsAg loss rate at 48 weeks. Compared with those who did not receive TCM, patients receiving combined TCM therapy showed a significantly higher HBsAg loss rate (OR = 3.83, 95% CI 2.13–6.87, P <0.001). Previous studies have indicated that TCM can exert anti-HBV effects directly or indirectly, with the latter being the primary mechanism. Direct mechanisms include inhibiting viral invasion, targeting HBV cccDNA, and blocking HBV transcription; indirect mechanisms involve multiple biological processes such as immunomodulation, cell death, autophagy, and oxidative stress (Ge et al., 2023).

A single-center retrospective cohort study demonstrated that the combination of TCM and PEG-IFNα-2b significantly reduced the quantitative level of HBsAg at 24 and 48 weeks, with a markedly superior efficacy compared to PEG-IFNα-2b alone (Liang et al., 2025). A real-world study by Lin Li et al. also confirmed that the combination of TCM and PEG-IFNα-2b exhibited significant advantages in reducing HBsAg levels, promoting HBV-DNA seroconversion, and improving the seroconversion rate of HBeAg (Lin et al., 2025). Additionally, the findings of Wang Zhanglin et al. further demonstrated that the compound TCM formula could effectively inhibit cellular HBsAg secretion in a time-dependent manner (Wang, 2017). TCM is characterized by multiple components, multi-targets, and multi-pathways, enabling it to exert anti-HBV effects through integrated mechanisms with a relatively low risk of drug resistance. However, its complex composition and the challenges in standardizing quality hinder full elucidation of its specific mechanisms of action, necessitating further in-depth research.

### 4.2 Baseline HBsAg level

Extensive studies have confirmed that baseline HBsAg quantitative level is a robust predictor of HBsAg loss (Zheng et al., 2019). The new Switch study, a multicenter, open-label, randomized controlled trial, demonstrated through subgroup analysis that patients with baseline HBsAg <1500 IU/mL had a significantly higher proportion achieving HBsAg <200 IU/mL by week 24 compared to those with baseline HBsAg ≥1500 IU/mL (58.7% vs. 10.9%, P<0.0001), and achieved markedly higher HBsAg loss rates by week 48 (11.5% vs. 1.6%). These findings further support low baseline HBsAg levels are an independent determinant of HBsAg loss (Hu et al., 2018). Another retrospective cohort study further validated HBsAg quantitative level as an independent predictor of HBsAg loss through multivariate regression analysis, identifying 72 IU/mL as the optimal cutoff; notably, 77% of patients with baseline HBsAg <100 IU/mL achieved clinical cure (Wen et al., 2024). Our results align with these findings, indicating that lower baseline HBsAg quantitative levels correlate with higher probabilities of achieving HBsAg loss by week 48 (OR = 0.32, 95% CI 0.25-0.42, P<0.001).

### 4.3 AST

Multivariate regression analysis in this study identified AST as an independent predictor of HBsAg loss. Higher baseline AST was associated with a lower probability of achieving HBsAg loss at week 48 (OR = 0.97, 95% CI: 0.95–0.99, P = 0.002). Previous studies have consistently demonstrated that both ALT and AST participate in immune regulation during HBV infection and serve as core biochemical markers for evaluating liver injury and treatment response to interferon. Most studies have shown that elevated transaminases during treatment reflect reactivated anti-HBV immunity, which correlates with superior antiviral outcomes (Zeng et al., 2025; Xie et al., 2022). Chen et al. also reported that patients with lower baseline HBsAg tended to have higher baseline transaminase levels, indicating stronger basal immune activity and a higher likelihood of responding to interferon therapy.

Although our study found that elevated baseline AST was unfavorable for HBsAg loss, this apparent discrepancy can be explained by biological differences between transaminase markers and inconsistencies in variable definitions across studies. ALT primarily reflects acute inflammatory immune activation triggered by HBV, whereas elevated baseline AST is more indicative of persistent hepatocyte injury and excessive chronic inflammatory burden. Severe liver damage may lead to immune exhaustion, blunting the immunomodulatory effect of interferon and reducing the chance of HBsAg loss. In line with our findings, a large real-world cohort study confirmed that baseline AST was markedly lower in patients who achieved HBsAg loss compared with non-responders [29.0 (22.1, 47.7) vs 34.8 (24.2, 59.3), P < 0.05], further supporting that a lower baseline AST favors HBsAg loss (Zhou et al., 2025).

Notably, most existing predictive models incorporate dynamic changes in AST at week 12 and week 24 of treatment, while the present analysis focused solely on a single baseline AST measurement without longitudinal on-treatment biochemical data. Differences in observation time points and variable types largely account for the heterogeneous predictive trends of AST across published studies. We plan to carry out prospective trials with serial AST monitoring throughout the treatment course to further clarify the time-phased predictive value of AST for PEG-IFN-induced HBsAg loss at different treatment stages.

### 4.4 History of ETV medication

NAs can effectively inhibit HBV replication by suppressing reverse transcription, but achieving HBsAg loss with NAs alone is challenging. Previous studies have shown that tenofovir analogs exhibit superior potential in inducing interferon-λ compared with ETV, likely due to their enhanced ability to activate the interferon signaling pathway. Additionally, TAF delivers higher concentrations of active drug to hepatocytes at lower doses than TDF (25 mg/day vs. 300 mg/day, respectively) (Choi et al., 2019; Tajiri et al., 2024). A prospective study comparing ETV and TAF in reducing serum HBsAg levels in patients with CHB demonstrated that TAF-treated patients showed significantly greater reductions in serum HBsAg levels than patients in the ETV group (P<0.001), although the mechanism underlying TAF’s enhanced HBsAg reduction requires further investigation (Uchida et al., 2022). Another retrospective study indicated that TDF was more effective than ETV in promoting HBeAg seroconversion and HBsAg loss (OR = 1.25), with most patients achieving HBsAg loss receiving TDF treatment (OR = 2) (Toygar Deniz et al., 2025). The results of this study are consistent with the aforementioned research. Multivariate regression analysis confirmed that a history of ETV use is an independent predictor of HBsAg loss. Compared with patients not receiving ETV therapy, those treated with ETV antiviral therapy exhibited a significantly lower HBsAg loss rate (OR = 0.33, 95% CI 0.17-0.63, P = 0.001).

Previous studies have demonstrated that factors such as age, sex, cirrhosis, HBV DNA levels, gamma-glutamyl transferase (GGT), hepatitis B genotypes (A, B, D), and dynamic changes in HBsAg and ALT during treatment may influence the loss rate of HBsAg after interferon therapy. A meta-analysis involving 7,913 patients identified younger age (OR = 0.951, P = 0.01), male sex (OR = 0.537, P = 0.001), low HBsAg levels (OR = 0.373, P<0.001), a greater decline in HBsAg at 12 weeks (OR = 6.689), a greater decline in HBsAg at 24 weeks (OR = 6.513), and elevated ALT at 12 weeks (OR = 3.622) as significant predictors of sustained virological response to PEG-IFNα (Jiang et al., 2024). Additionally, studies have reported a significant correlation between baseline GGT levels and HBsAg loss rate (AUC = 0.607). Lower GGT levels indicate a significant reduction in hepatic inflammation and cholestasis, suggesting relatively better hepatocyte function, which may more effectively support the antiviral immune response of PEG-IFN (Kong et al., 2025). However, in this study, no significant correlation was observed between sex, age, ALT, HBV DNA, and HBsAg loss rate, possibly due to factors such as study population characteristics, regional differences, sample size, and follow-up duration. Furthermore, this study did not include analyzes of variables such as GGT, treatment process dynamic indicators, HBV genotypes (A, B, D), and cirrhosis status, leaving room for further refinement.

This study constructed a predictive model for achieving HBsAg loss through multivariate logistic regression analysis, identifying combined TCM therapy, baseline HBsAg quantitative level, AST level, and ETV medication history as key factors influencing HBsAg loss. The model demonstrated excellent discriminant efficacy and fit in both training and validation sets, with AUC values of 0.884 and 0.866, respectively, indicating high reliability in predicting HBsAg loss rates in CHB patients treated with PEG-IFNα-2b. DCA results suggested that this model could help screen patients more likely to achieve HBsAg loss in clinical practice, thereby guiding the selection of more suitable patients for intensive antiviral therapy while reducing unnecessary interventions for patients with lower loss rates, reflecting the concept of precision medicine. Compared with previous studies (Tan et al., 2024; Dong et al., 2025; Fu et al., 2025b; Kong et al., 2025; Yan et al., 2025), the innovation of this study lies in the first inclusion of combined TCM therapy in the predictive model, which was confirmed to significantly improve HBsAg loss rates, though its underlying mechanisms require further exploration.

This study possesses several strengths. First, the established predictive model is a non-invasive, convenient and safe risk assessment tool. It can effectively assist clinicians in screening eligible candidates for PEG-IFN therapy and optimizing individualized antiviral strategies, and is also generalizable to risk evaluation scenarios for various diseases. Second, the sample size of this study was adequate, and both the training and validation cohorts met rigorous statistical requirements for model development and validation, ensuring stable and reliable predictive performance. Nevertheless, several inherent limitations of this study should be acknowledged.

First, the external validation of the predictive model was restricted by the number of participating centers. To compensate for the lack of an independent third-party external validation cohort, one-way cross-center validation was performed to assess model stability. The two participating centers had unbalanced sample distribution: 85% of participants were recruited from TCM Center A and only 15% from Western medicine Center B. To avoid biased regression fitting caused by training the model on the small Center B cohort, all variable screening and model derivation were completed exclusively using data from the well-powered Center A, while all records from Center B were fully isolated throughout modeling to eliminate data leakage. When validated in the independent Center B cohort, the model achieved an AUC of 0.874 (95% CI: 0.804–0.945), which suggested favorable discriminative performance across different types of medical institutions (**Supplementary Figure 1**). Nevertheless, the model was established based solely on data from two centers, and its generalizability to patients from hospitals nationwide requires further verification in multicenter external cohorts.

Second, the analytical population was defined using a per-protocol set, which may lead to selection bias. A per-protocol analysis was adopted in this study. Only patients who completed full 48-week Peg-IFN treatment with complete week-48 serological records were enrolled. Subjects who discontinued treatment early due to early HBsAg loss, adverse drug reactions, early virological non-response or loss to follow-up were excluded, which inevitably introduced selection bias and limited the extrapolation of the model to all treatment-naive CHB patients initiating interferon therapy. Future research will adopt an intention-to-treat design including all baseline enrollees and conduct multicenter external validation to mitigate this bias.

Finally, we recognize several limitations related to the TCM herbal intervention in this retrospective real-world analysis. First, the distribution of TCM syndromes among enrolled patients was severely imbalanced. Specifically, 68% of participants diagnosed with liver-stagnation and spleen-deficiency syndrome treated primarily with modified Xiaoyao San. In contrast, the sample sizes of other syndromes such as liver-gallbladder damp-heat and spleen-kidney yang deficiency were relatively small, which introduced syndrome-related confounding and prevented stratified analyses comparing the therapeutic effects of TCM across distinct syndromes. Second, although the proportion of patients receiving combined TCM therapy was comparable between the two centers and interaction tests confirmed no center-based difference in the predictive effect of TCM (P<0.05), inherent discrepancies in clinical management strategies and follow-up protocols across hospitals could not be fully eliminated in real-world practice, leaving the possibility of residual center-associated bias. Third, the specific biological and immunological mechanisms responsible for the synergistic effect of TCM herbs on improving interferon efficacy remain unclear in this study. Based on the demographic characteristics of the present cohort, we plan to conduct a prospective single-syndrome real-world study exclusively enrolling CHB patients with liver-stagnation and spleen-deficiency syndrome. Participants from both TCM and Western medicine hospitals will be evenly stratified to balance inter-center clinical differences. Standardized regimens regarding TCM treatment duration and herbal modification rules will be uniformly implemented to further minimize confounding bias derived from TCM syndromes and study centers. This design will allow more precise evaluation of the real-world clinical benefits of modified Xiaoyao San combined with interferon for achieving functional cure of chronic hepatitis B, and further explore the potential immune and molecular mechanisms underlying the synergistic therapeutic effects of integrated TCM and Western medicine therapy.

## Data Availability

The datasets generated or analyzed during the current study are available from the corresponding author on reasonable request.
