# Supplementary material for: Construction and validation of a predictive model for HBsAg loss in chronic hepatitis B patients treated with Peg-IFNα-2b
Source: Front Cell Infect Microbiol. 2026 Jul 28;16:1865116. doi: 10.3389/fcimb.2026.1865116 (PMC13457193; doi:10.3389/fcimb.2026.1865116)
Supplement: Supplementary file 1 [file DataSheet1.docx]

**Supplementary Figure 1. One-way cross-center validation of the prediction model developed in Center A and independently tested in Center B.**

ROC curves, calibration curves, and decision curve analysis of the predictive model. A, ROC curve for the center B. B, Calibration curves for the center B. C, DCA for the center B.

Abbreviations: ROC, receiver operating characteristic; DCA, decision analysis curve

**Supplementary Table 1. Comparison of TCM treatment status and TCM syndrome distribution between two research centers**

| variable | Total patients | Center A | Center B | P value |
| --- | --- | --- | --- | --- |
|  | N=859 | N= 731 | N= 128 |  |
| Combined traditional Chinese medicine therapy | 399 (46.4%） | 346 (47.3%) | 53(41.4%) | 0.215 |
| TCM syndrome classification, n (%) |  |  |  | 0.935 |
| Liver-stagnation and spleen-deficiency | 273 (68.4%) | 237 (68.5%) | 36 (67.9%) |  |
| Liver-kidney yin deficiency | 53 (13.3%) | 45 (13.0%) | 8 (15.1%) |  |
| Blood stasis obstructing collaterals | 41 (10.3%) | 35 (10.1%) | 6 (11.3%) |  |
| Liver-gallbladder damp-heat | 17 (4.3%) | 15 (4.3%) | 2 (3.8%) |  |
| Spleen-kidney yang deficiency | 15 (3.7%) | 14 (4.0%) | 1 (1.9%) |  |

Data are presented as n (%) for categorical variables. Center A represents the traditional Chinese medicine hospital, and Center B represents the Western general hospital. P values were calculated by the Chi-square test.
